# Characteristics of p-Type Conduction in P-Doped MoS_2_ by Phosphorous Pentoxide during Chemical Vapor Deposition

**DOI:** 10.3390/nano9091278

**Published:** 2019-09-07

**Authors:** Jae Sang Lee, Chang-Soo Park, Tae Young Kim, Yoon Sok Kim, Eun Kyu Kim

**Affiliations:** Department of Physics and Research Institute of Natural Sciences, Hanyang University, Seoul 04763, Korea (J.S.L.) (C.-S.P.) (T.Y.K.) (Y.S.K.)

**Keywords:** chemical vapor deposition, P_2_O_5_, p-type conduction, P-doped MoS_2_

## Abstract

We demonstrated p-type conduction in MoS_2_ grown with phosphorous pentoxide via chemical vapor deposition (CVD). Monolayer MoS_2_ with a triangular shape and 15-µm grains was confirmed by atomic force microscopy. The difference between the Raman signals of the A_1g_ and E^1^_2g_ modes for both the pristine and P-doped samples was 19.4 cm^−1^. In the X-ray photoelectron spectroscopy results, the main core level peaks of P-doped MoS_2_ downshifted by about 0.5 eV to a lower binding energy compared to the pristine material. Field-effect transistors (FETs) fabricated with the P-doped monolayer MoS_2_ showed p-type conduction with a field-effect mobility of 0.023 cm^2^/V⋅s and an on/off current ratio of 10^3^, while FETs with the pristine MoS_2_ showed n-type behavior with a field-effect mobility of 29.7 cm^2^/V⋅s and an on/off current ratio of 10^5^. The carriers in the FET channel were identified as holes with a concentration of 1.01 × 10^11^ cm^−2^ in P-doped MoS_2_, while the pristine material had an electron concentration of 6.47 × 10^11^ cm^−2^.

## 1. Introduction

Recently, various studies have analyzed two-dimensional (2D) materials, such as graphene, MoS_2_, and WSe_2_, because of their critical properties and abundant potential for use in optical and electrical applications [1,2,3]. Graphene has a zero band gap structure, but has not been able to replace semiconductor-based devices [4,5]. Additionally, layered transition metal dichalcogenides (TMDs) such as MoS_2_ and WSe_2_ have received enormous attention as promising materials and layer structures, in which transition metals are sandwiched between two chalcogen atom layers by a covalent force. Moreover, there are Van der Waals (VdW) forces interacting in individual layers, which make exfoliation easily. Interestingly, these materials have a unique property; their band gap structure varies depending on the thickness. In the case of MoS_2_, the band gap of a monolayer has a direct band gap of 1.8 eV, while a few layers of MoS_2_ and bulk MoS_2_ have an indirect band gap structure with a band gap of about 1.2 eV [6].

The chemical vapor deposition (CVD) method has several advantages compared to other methods, such as mechanical and liquid exfoliation methods [7,8]. The disadvantages of the Scotch tape-based mechanical method are its difficulty in controlling the flake thickness, size, and uniformity, which makes it inappropriate for large-scale applications. The liquid method still needs to be developed for applications, while the CVD method has been used to prepare ultrathin monolayers or few-layer MoS_2_ films over large areas [9]. Transistors have been fabricated via CVD growth of monolayer MoS_2_. These have been reported to exhibit good properties, including a high on/off current ratio and high mobility [10]. To realize detailed applications, this method needs to be able to produce a junction composed of n- and p-type materials. Although there have been many challenges to p-type doping of MoS_2_ using niobium (Nb) or phosphorous (P) atoms [11,12], and it remains difficult to successfully dope ultrathin MoS_2_. According to a previous report, P atoms seem to be the most suitable acceptors among group V elements [13].

In this paper, we report on the CVD growth and characteristics of monolayer MoS_2_ with and without the addition of phosphorous pentoxide (P_2_O_5_) powder. The thickness and grain size of the MoS_2_ layer were measured using non-contact-mode atomic force microscopy (AFM) and Raman spectroscopy. To confirm the electrical characteristics of MoS_2_, back-gated field-effect transistors (FETs) were fabricated. The p-type conduction from monolayer MoS_2_ grown with P_2_O_5_ powder was confirmed and compared to pristine MoS_2_ with n-type behavior.

## 2. Experimental

To synthesize an MoS_2_ layer by the CVD method, molybdenum trioxide (MoO_3_, CERAC Inc, Milwaukee, WI, USA) powder with 99.999% purity as a precursor material and sulfur (iTASCO Inc, Seoul, Korea) powder of 99.999% purity as a reactant material were used. For p-type doping of MoS_2_ in this experiment, 98.99% purity P_2_O_5_ (SIGMA-ALDRICH, St. Louis, MO, USA) powder was added as a dopant material. SiO_2_/Si substrates (2 × 2 cm^2^) with a SiO_2_ thickness of 270 nm and three alumina boats were used. The alumina boats were filled with 10 mg of MoO_3_ powder, 300 mg of S powder, and 1 mg of P_2_O_5_ powder, respectively. During CVD growth of MoS_2_, the furnace was heated to 750 °C with a heating rate of 30 °C/min under argon gas flowing at 100 sccm. The role of argon gas was to transport S and P_2_O_5_ when they were vaporized. During the growth of MoS_2_, the gas flow and furnace temperature were kept constant for 30 min, and then the furnace was quickly cooled down to room temperature.

The MoS_2_ thickness and grain size were analyzed by using non-contact-mode atomic force microscopy (AFM) (XE-100, Park’s Systems, Seoul, Korea) and optical microscopy. X-ray photoelectron spectroscopy (XPS) (K-Alpha+, Thermo Fisher Scientific, Waltham, MA, USA) under ~4 × 10^−10^ Torr and Raman spectroscopy (NRS-3100, JASCO, Tokyo, Japan) with a λ = 532 nm laser were measured at room temperature to identify the doping characteristics. To confirm the electrical characteristics of doped monolayer MoS_2_, back-gated FETs were fabricated. In this process, photolithography was used for patterning source and drain electrodes of Ni/Au (5 nm/50 nm) metals.

## 3. Results

**Figure 1:**
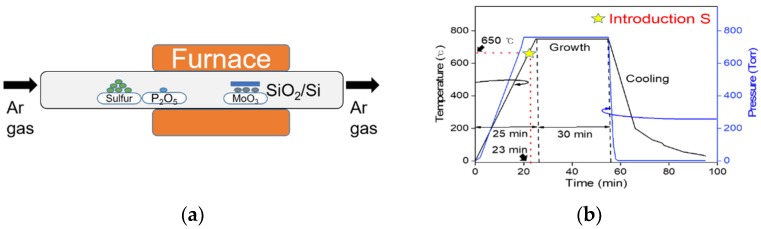
(**a**) Schematic diagram of the chemical vapor deposition (CVD) process for the monolayer MoS_2_ synthesis and in situ P doping with P_2_O_5_ powder. (**b**) Temperature profile of the reaction furnace and pressure in the quartz tube as a function of the processing time.

Figure 1a shows a simplified schematic diagram for the synthesis of MoS_2_, with and without phosphorus doping, using the CVD system. Here, MoO_3_ powder was placed in the middle of the furnace, slightly away from the S and P_2_O_5_ powders. The P_2_O_5_ powder for P doping was located about 7.5 cm from the MoO_3_ powder. The ratio of S to Mo atoms is an important point for growing monolayer MoS_2_ flakes. We used a face-down substrate approach, where the SiO_2_ substrate is positioned vertically facing the MoO_3_-containing alumina boat. Unlike previous doping studies that used a two-furnace system [14,15,16], this method used in situ doping with a one-furnace CVD system. The CVD process for MoS_2_ growth can be divided into two steps: Nucleation and growth. Figure 1b shows the temperature profile of the reaction furnace and pressure variation in the quartz tube with Ar gas flow, respectively, as a function of time. In this figure, S atoms are introduced at 650 °C which is 100 °C lower than the growth temperature (750 °C). When S atoms are introduced, the growth of MoS_2_ starts and then monolayer MoS_2_ flakes appear [17].

**Figure 2:**
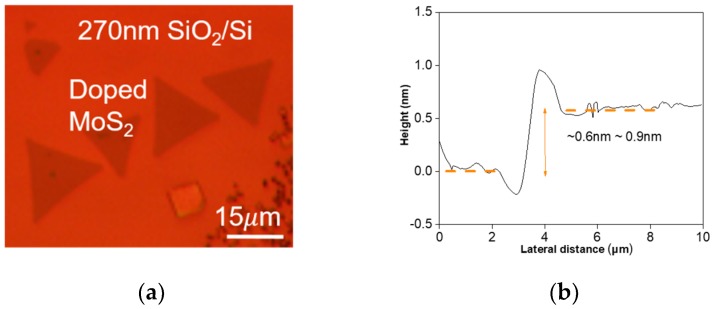

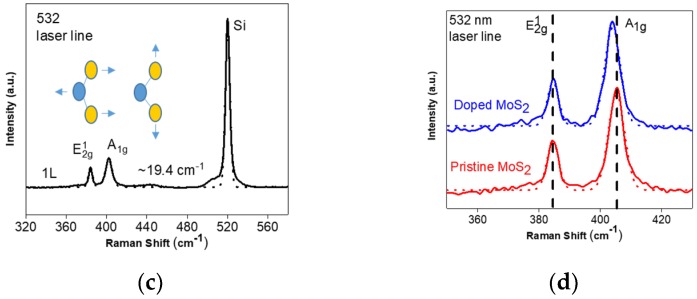
(**a**) Optical microscope image, (**b**) AFM height profile, and (**c**) Raman spectroscopy results using a laser with an excitation wavelength of 532 nm for a monolayer of CVD-grown MoS_2_ flakes. (**d**) The two Raman modes for the pristine and doped monolayer MoS_2_ flakes.

Figure 2a shows an optical microscopy image of P-doped MoS_2_ grown via CVD. Here, the MoS_2_ layers grown on the SiO_2_/Si substrate under a sufficient S atmosphere were observed to have a triangular shape [17,18]; this is the same shape as pristine MoS_2_. The grain size of doped MoS_2_ on the SiO_2_/Si substrate was about 15 µm. To confirm the formation of a monolayer of P-doped MoS_2_, Raman spectroscopy and AFM measurements were performed, as shown in Figure 2b,c, respectively. The thickness of an MoS_2_ flake measured by AFM was about 0.6 nm to 0.9 nm; this layer thickness is the same as a previous result [10]. This measurement value corresponds to the interlayer spacing of a monolayer of S-Mo-S bonding in the MoS_2_ crystal. Two characteristic Raman peaks, i.e., E^1^_2g_ and A_1g_ from in-plane and out-of-plane modes, respectively, were measured by a laser with an excitation wavelength of 532 nm at room temperature, as shown in Figure 2c. The in-plane E^1^_2g_ mode presents the vibration of one Mo atom and two S atoms in opposite directions, while the out-of-plane A_1g_ mode vibrates only S atoms in opposite directions (as shown in the inset of Figure 2c). From reported results that describe the dependence of the Raman peaks on the number of layers [19,20], we know that the difference between two Raman peaks depending on the number of MoS_2_ layers is larger than 20 cm^−1^ for thicknesses above a bilayer (2 L). As shown in Figure 2d, Raman peaks from P-doped MoS_2_ were located at 384.5 cm^−1^ (E^1^_2g_ mode) and 403.9 cm^−1^ (A_1g_ mode). On the other hand, the E^1^_2g_ and A_1g_ signals of the pristine monolayer MoS_2_ were observed at around 384.6 cm^−1^ and 405 cm^−1^, respectively. The difference between the two Raman modes for P-doped and pristine MoS_2_ (Figure 2d) appear to be about 19.4 cm^−1^ and 20 cm^−1^, respectively; these values indicate a single layer of MoS_2_.

Here, the Raman signal peak of the A_1g_ mode was found to be shifted by about 1.1 cm^−1^, while the signal peak of the E^1^_2g_ mode was almost unchanged. Azcatl et al. reported that a strain induced by dopants can generate contractions of the MoS_2_ lattice structure [21]; this phenomenon occurs due to the longer bond length of Mo-S atoms than that of Mo-P atoms. It was also reported that the A_1g_ mode is often more influenced by doping effects than other modes (e.g., the E^1^_2g_ mode); this is due to its strong coupling with electrons [22,23]. Therefore, the Raman active signal with A_1g_ has a shift larger than the other Raman active signal because this peak of the Raman mode is quite sensitive to the doping effect. We confirmed that the Raman shifts in Figure 2d agreed with previous results [24]. The full width at half maximum (FHWM) of the E^1^_2g_ peak was investigated to characterize the crystalline quality of MoS_2_ obtained by the CVD synthesis method. The FWHM result of the CVD-grown monolayer flake is 3.8 cm^−1^, which is similar to a recently reported value of a CVD-synthesized single-layer flake [18]. 

**Figure 3:**
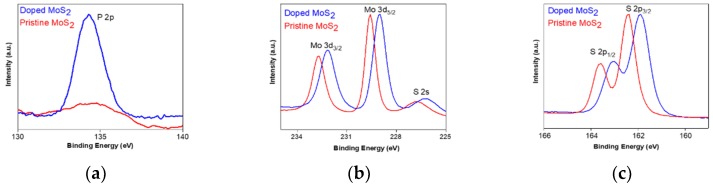
XPS spectra of (**a**) P 2p, (**b**) Mo 3d, and (**c**) S 2p peaks in the pristine and doped MoS_2_. These results indicate that the peaks of each core level are downshifted in the doped MoS_2_ flake.

The energy peaks appearing in XPS were also analyzed to confirm the doping properties in monolayer MoS_2_ crystals. Figure 3a–c show the comparative XPS core level analyses of pristine and doped monolayer MoS_2_. In Figure 3a, the P 2p binding energy peak, which clearly appears only at 134.3 eV, is associated with a doped flake feature. It is worth mentioning that the existence of this peak provides apparent evidence that P_2_O_5_ takes its position before the introduction of S. In addition, the Mo 3d and S 2p core levels indicated that the phenomenon causes a uniform shift of 0.5 eV, from 229.6 eV to 229.1 eV and from 162.4 eV to 161.9 eV, respectively (Figure 3b,c). That is, each peak moved toward a lower binding energy after P-doping, which is very similar to the reported results for Nb-doped MoS_2_ [24]. This study reported that the Fermi level (E_F_) of pristine MoS_2_ is located close to the conduction band (E_c_) edge, while an Nb-doped p-type sample has a Fermi level near the valence band edge. The work function and electron affinity of the pristine monolayer MoS_2_ is 5.1 eV and 4.28 eV, respectively [25]. The pristine MoS_2_ Fermi level is 0.82 eV, which is the E_c_–E_F_ result, and the doping sample Fermi level is 1.32 eV, 0.82 + ΔBE (measured from XPS data). Therefore, it is suggested that doping with P_2_O_5_ leads to a downshift in the Fermi level of about 0.5 eV, close to the valence band maximum.

**Figure 4:**
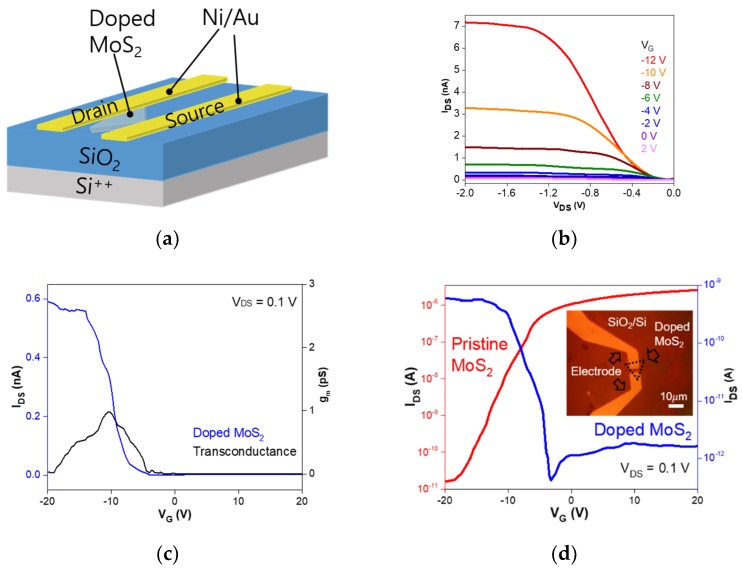
(**a**) Schematic of field-effect transistors (FETs) with a channel length of 3 nm and a channel width of 10 nm. (**b**) I_DS_–V_DS_ curves of a P-doped monolayer MoS_2_ FET with different gate voltages_._ (**c**,**d**) Linear and log scales of the transfer characteristics as a function of the gate voltage for FETs with pristine and P-doped MoS_2_ channels, respectively.

Figure 4a,b show the fabricated back-gate FET schematic with a channel length of 3 µm and a channel width of 10 µm, as well as the I_DS_–V_DS_ curve of an FET based on P-doped monolayer MoS_2_, respectively.

Here, Ohmic metals of Ti and Ni were used for n-type pristine and p-type P-doped MoS_2_ FETs, respectively, to match the metal work functions [26]. Figure 4c shows the transfer characteristics of these devices fabricated on pristine and P-doped monolayer MoS_2_ flakes. The inset image of Figure 4d is the optical microscopy image of MoS_2_ FETs which was fabricated on the doped monolayer MoS_2_. The pristine MoS_2_ FETs demonstrates n-type conduction with a high on/off current ratio of ~10^5^ [27]. The threshold voltage V_T_ value extracted by the linear extrapolation method was about −8.1 V. In the case of P-doping, the transfer curve indicated p-type conduction with an on/off current ratio of ~10^3^, and the V_T_ was −6.9 V at a drain-source voltage of 0.1 V. The field-effect mobilities of these FETs were calculated by the following relation:

*µ*= (dI_DS_/dV_BG_) × [L/C_ox_ WV_DS_], (1)

where L and W are the channel length and width, respectively. The back-gate capacitance (C_ox_ = ε_0_ε_r_/d) was ~1.28 × 10^−8^ F/cm^2^, where ε_0X_ is the dielectric constant and d is the thickness of silicon oxide. Using the transconductance value obtained by the relation of g_m_ = dI_DS_/dV_BG_, the field-effect mobilities were determined to be about 29.7 cm^2^/V⋅s and 0.023 cm^2^/V⋅s for the pristine and P-doped MoS_2_ FETs, respectively. The carrier concentration in the FET channel could also be estimated by using the following relation:

n = C_ox_ (V_BG_ − V_T_)/e, (2)

where e is the electron charge [28]. The electron concentration in pristine MoS_2_ was 6.47 × 10^11^ cm^−2^, whereas the hole concentration in P-doped MoS_2_ was 1.01 × 10^11^ cm^−2^. Based on these results, the complete p-type conduction of MoS_2_ with the addition of P_2_O_5_ was demonstrated in this study.

## 4. Conclusions

We have demonstrated the p-type conduction of P-doped MoS_2_ by P_2_O_5_ via a CVD process. Based on AFM and Raman measurements, pristine and P-doped MoS_2_ were confirmed to have monolayer thickness with grain sizes in the order of 15 µm. From XPS data, it was suggested that the Fermi level of P-doped MoS_2_ shifted by about 0.5 eV toward the valence band compared to the pristine MoS_2_. FETs with P-doped monolayer MoS_2_ showed p-type conduction with a field-effect mobility of 0.023 cm^2^/V⋅s and an on/off current ratio of 10^3^, while pristine MoS_2_ FETs had n-type behavior with a field-effect mobility of 29.7 cm^2^/V⋅s and an on/off current ratio of 10^5^. The carriers in the FET channel were identified to be holes with a concentration of 1.01 × 10^11^ cm^−2^ in P-doped MoS_2_ and electrons with a concentration of 6.47 × 10^11^ cm^−2^ in the pristine material. This phosphorous doping technique should be applicable to other TMD materials.
